# Clinical and Biological Significances of a Methyltransferase-Related Signature in Diffuse Glioma

**DOI:** 10.3389/fonc.2020.00508

**Published:** 2020-04-20

**Authors:** Ying Zhang, Yuqing Liu, Hanjie Liu, Zheng Zhao, Fan Wu, Fan Zeng

**Affiliations:** ^1^Department of Molecular Neuropathology, Beijing Neurosurgical Institute, Capital Medical University, Beijing, China; ^2^Chinese Glioma Genome Atlas Network (CGGA) and Asian Glioma Genome Atlas Network (AGGA), Beijing, China

**Keywords:** glioma, methyltransferase, signature, prognosis, risk score

## Abstract

Methylation of DNA, RNA or protein is a reversible modification. The proteins and genes that regulate this modification can be a candidate target for tumor therapy. However, the characteristics of methyltransferase related genes in glioma remain obscure. In this study, we systematically analyzed the relationship between methyltransferase-related genes expression profiles and outcomes in glioma patients based on The Cancer Genome Atlas and Chinese Glioma Genome Atlas RNA sequencing datasets. Consensus clustering identified two robust groups with significantly different pathological features and prognosis. Then a methyltransferase-related risk signature was built by a Cox proportional hazards model with elastic net penalty. Moreover, the risk score is associated with patients' clinical and molecular features and can be used as an independent prognostic indicator for patients with glioma. Furthermore, genes associated with the high-risk group were involved in various aspects of the malignant progression of glioma via Gene Ontology analysis and Gene Set Enrichment Analysis. In summary, our study identified a methyltransferase-related risk signature for predicting the prognosis of gliomas.

## Introduction

In the central dogma of molecular biology, genetic information flows from DNA, RNA to proteins (1). Reversible epigenetic modifications can influence gene expression without altering the DNA sequence, and thus determine cell differentiation and development. DNA modifications, RNA modifications, protein modifications, and nucleosome remodeling are all in the field of epigenetics. These modifications comprise methylation, acetylation, phosphorylation, ribosylation, sumoylation, parylation, citrullination, ubiquitylation, etc (2). Among them, methylation is widely studied and is defined as an important and extensive epigenetic modification. Depending on the substrate, this modification can be divided into DNA, RNA or protein methylation, which are mediated by corresponding methyltransferase.

In mammals, DNA methyltransferases (DNMTs) maintain DNA methylation via transferring methyl group to cytosines of CpG dinucleotide islands. Aberant DNA hypermethylation of tumor suppressor gene promoter region results in gene silencing, which subsequently leads to dysregulation of diverse signaling pathways associated with human malignancies (3). O-6-methylguanine-DNA methyltransferase (MGMT) is involved in cellular defense against the toxicity of alkylating agents such as temozolomide (TMZ) (4). Patients with glioblastoma (GBM) containing a methylated *MGMT* promoter can benefited from TMZ therapy (5). *MGMT* promoter status has been identified as a biomarker for TMZ response in GBM patients.

Approximately 150 chemical modifications have been identified in eukaryotic cellular RNAs. The spectrum of major physiological mRNA methylation marks comprises methylations of adenosine to form N^6^-methyladenosine (m^6^A), N^1^-Methyladenosine (m^1^A) and N^6^, 2′-O-dimethyladenosine (m^6^A_m_), as well as cytosine methylation to 5-methylcytosine (m^5^C) and its oxidation product 5-hydroxymethylcytosine (hm^5^C) (6, 7). Among them, m^6^A is the most prevalent form of internal mRNA methylation. RNA methylation has diverse effects on RNA metabolism, including RNA processing, RNA splicing, mRNA export, mRNA translation, and decay (7). The m^6^A mRNA modification is critical for glioblastoma stem cells (GSCs) self-renewal and tumorigenesis (8). Knockdown of *METTL3* or *METTL14*, key components of the RNA methyltransferase complex, dramatically promotes human GSC growth, self-renewal, and tumorigenesis (8). In contrast, overexpression of *METTL3* or inhibition of the RNA demethylase *FTO* suppresses GSC growth and self-renewal (8). Moreover, the m^6^A demethylase ALKBH5 is highly expressed in GSCs, and silencing ALKBH5 suppresses the proliferation of patient-derived GSCs (9).

In eukaryotes, most protein methylation is implemented by two widely defined enzyme families: lysine methyltransferases (KMTs) and protein arginine methyltransferases (PRMTs), which modify the ε amino group of lysine (K) and the guanidinium group of arginine (R), respectively (10). In humans, over 4,000 K and R methylation sites have been identified, but the biological consequence of most is unknown (10). Histone proteins are a major and well-studied substrate of protein methyltransferases (PMTs). It is believed that methylation of K or R residues in the tail of histones largely decides the chromatin configurations, thus determining gene expression, cell fate and genomic stability (11). EZH2 is a catalytic component of polycomb repressive complex 2 (PRC2), which is responsible for the trimethylation of histone 3 on lysine 27 (H3K27me3) and induces gene silencing (12). EZH2 is a negative independent prognostic factor and exhibits tumor promoting activity in GBM (13). Meanwhile, methylation of several non-histone proteins participated in tumor-associated signaling pathways, including p53 (14, 15), RB1 (16, 17), NF-κB (18, 19), STAT3 (20), etc. EZH2 binds to and methylates STAT3, leading to enhanced STAT3 activity by increased tyrosine phosphorylation of STAT3 (20). The EZH2-STAT3 interaction preferentially occurs in GSCs and promotes its tumorigenicity (20).

Glioma is the most common primary malignant brain tumors, characterized by high recurrence rates, short survival time, high mortality, and treatment difficulties (21). Currently, the clinical outcomes for glioma patients are still poor even after standard treatments, including surgery, chemotherapy and radiation (22). An in-depth understanding of the molecular landscape of diffuse glioma reveals its characteristic genetic and epigenetic features and clarifies their pathogenic evolution (23–26). In 2016 WHO classification, mutations in the epigenetic modulator genes isocitrate dehydrogenase 1 or 2 (IDH1 or IDH2) and codeletion of chromosomal arms 1p/19q (1p/19q codel) have become key biomarkers for glioma classification (27, 28). It emphasized the role of genetic and epigenetic alterations as a driving force for glioma evolution. Methyltransferase-related genes play an important role in epigenetic regulation, including DNA, RNA, histone methylation. Some of striking members, such as EZH2 (13), FTO (8) and ALKBH5 (9), have been reported to play oncogenic roles in glioma genesis. However, the expression pattern of methyltransferase complex genes in glioma patients and its prognostic value remain to be further elucidated.

In this study, we systematically analyzed the characteristics of the methyltransferase-related genes in glioma based on TCGA (*n* = 601) and CGGA (*n* = 309) RNA sequencing datasets. We found that the methyltransferase genes could classify the glioma patients with significantly different clinical and molecular characteristics. And a risk signature with twelve methyltransferase-related genes was designed to predict prognosis of glioma patients.

## Materials and Methods

### Data Collection

The TCGA RNA sequencing (RNA-seq) dataset and corresponding clinical and molecular information, such as gender, age, grade, subtype, *IDH* status, 1p/19q status, *MGMT* promoter status, *EGFR* status, and survival information, were downloaded from TCGA database (http://www.cancergenome.nih.gov/) as training cohort. Similarly, the CGGA RNA-seq database (http://www.cgga.org.cn) with 309 glioma samples were obtained as validation cohort.

### Consensus Clustering

Methyltransferase-related genes (GO_METHYLOSOME and GO_METHYLTRANSFERASE_COMPLEX) were obtained from Molecular Signature Database v5.1 (MSigDB) (http://www.broad.mit.edu/gsea/msigdb/) (29). After overlapping with genes in the TCGA and CGGA RNA-seq data sets, 89 and 84 methyltransferase-related genes, respectively, remained. Then we carried out consensus clustering with the R package “ConsensusClusterPlus.” The optimal number of subgroups was evaluated using cumulative distribution function (CDF) and consensus matrices (30).

### Gene Signature Identification and Risk Score Construction

The prognostic value of methyltransferase-related genes in TCGA training cohort was computed by a univariate Cox regression analysis. *P* ≤ 0.05 is considered statistically significant. After that, the least absolute shrinkage and selection operator (LASSO) method was used to identify gene signature and obtain their respective coefficients (Coeff) value. According to the following formula, the risk score for each patient was calculated in TCGA training and CGGA validation cohorts.

$$\begin{array}{l} \text{Risk score}=\overset{n}{\underset{i=1}{\sum }}\text{exp}{\text{r}}_{\text{gene}\left(\text{i}\right)}\times {\text{Coeff}}_{\text{gene}\left(\text{i}\right)} \end{array}$$

### Statistical Analysis

Patients in TCGA training and CGGA validation cohorts were separated into high-risk and low-risk groups by using the median risk score as a threshold. Kaplan–Meier survival analysis and 2-sided log-rank test were used to calculate the overall survival (OS) differences between stratified groups. Univariate and multivariate Cox regression analysis were used to determine independent prognostic factors, including gender, age at diagnosis, WHO grade, *IDH* status, 1p/19q status, *MGMT* promoter status, *EGFR* status, and risk score. ROC curve analysis was used to predict OS with R package “pROC.” Student's *t*-test and chi-square test were adopted to detect differences in pathology and molecular characteristics between different patient groups. All statistical analyses were conducted by SPSS 16.0 (Armonk, NY, USA) or R software, and *P* ≤ 0.05 was regarded as statistically significant.

### Gene Ontology (GO) and Gene Set Enrichment Analysis (GSEA)

Biological process, cell component and protein-protein interactions among genes in the risk signature were analyzed by the STRING database (https://string-db.org/) (31). Pearson correlation analysis using R language to calculate genes that are positively and negatively correlated with risk scores in TCGA and CGGA datasets (|R| > 0.5, *P* < 0.0001). GO and KEGG pathway analysis were performed for functional annotation of the significantly correlated genes via the online Database for Annotation, Visualization and Integrated Discovery (DAVID, https://david.ncifcrf.gov/). Gene set enrichment analysis (GSEA, http://software.broadinstitute.org/gsea/index.jsp) was performed to identify gene sets of statistical difference between low-risk and high-risk groups.

## Results

### Classification of Gliomas Based on Methyltransferase-Related Genes

**Figure 1:**
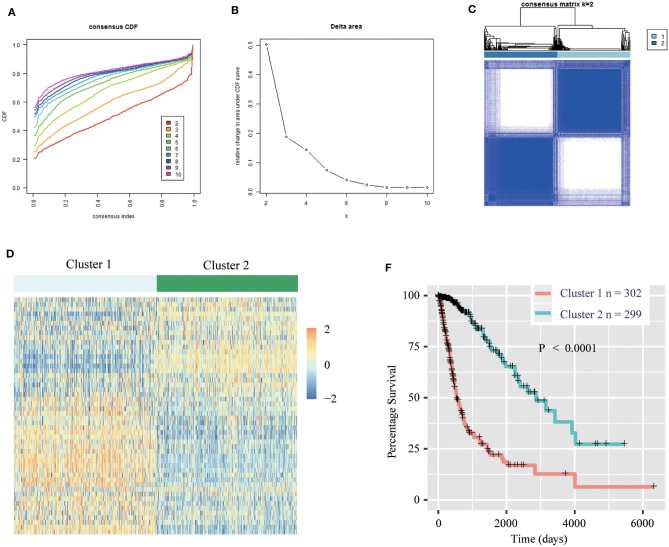
Methyltransferase-related genes could classify the clinical and molecular features of gliomas. **(A)** Consensus clustering cumulative distribution function (CDF) for *k* = 2 to *k* = 10. **(B)** Relative change in area under CDF curve according to various k values. **(C)** Consensus clustering matrix of 601 samples from TCGA dataset for *k* = 2. **(D)** Heat map of two clusters defined by the top 50 variable expression genes. **(E)** Survival analysis of patients in Cluster 1 and 2 in TCGA cohort.

We obtained the methyltransferase-related gene expression profiling of 601 samples from the TCGA RNA-seq dataset, and the top 50 variable expression genes have been selected. Consensus clustering of the 601 samples could divided patients into two significantly different clusters (Figures 1A–C), and the heatmap of the two clusters has been shown in Figure 1D. We found that consensus clustering could make significant differences in the clinical and molecular characteristics of the two glioma clusters (Table 1). Cluster 1 patients were strikingly correlated with older age at diagnosis (64.24%, *P* < 0.0001), high grade (49.01%, *P* < 0.0001), classical or mesenchymal subtypes (56.95%, *P* < 0.0001), *IDH* wildtype (68.87%, *P* < 0.0001), 1p/19q non-codel (91.40%, *P* < 0.0001), *MGMT* promoter unmethylation (44.07%, *P* < 0.0001), and *EGFR* amplification (34.34%, *P* < 0.0001) by Chi-square test. Compared with patients in cluster 2, glioma patients in cluster 1 showed a shorter survival time (Log-rank, *P* < 0.0001, Figure 1E). Then, the similar results were shown in Figure S1 and Table S1 based on the CGGA RNA-seq dataset (*n* = 309). These results demonstrated that methyltransferase-related genes were associated with the malignancy and prognosis of diffuse gliomas.

**Table 1:** Characteristics of patients in cluster 1 and 2 in TCGA cohort.

**Characteristics**	***n***	**Cluster 1**	**Cluster 2**	***P-*value**
**Total Cases**	601	302	299	
**Age**				
**<**49	320	108	212	** <0.0001**
≥49	281	194	87	
**Gender**				
Male	351	178	173	0.8524
Female	250	124	126	
**Grade**				
II	211	52	159	**<0.0001**
III	236	102	134	
IV	154	148	6	
**Subtype**				
Neural	36	9	27	**<0.0001**
Proneural	381	121	260	
Classical	149	143	6	
Mesenchymal	35	29	6	
***IDH*** **status**				
Mutant	374	94	280	**<0.0001**
Wildtype	227	208	19	
**1p/19q status**				
Codel	149	8	141	** <0.0001**
Non-codel	223	85	138	
NA	229	209	20	
***MGMT*** **promoter status**				
Unmethylated	147	119	28	** <0.0001**
Methylated	420	151	269	
NA	34	32	2	
***EGFR*** **status**				
Amplification	106	102	4	** <0.0001**
No amplification	487	195	292	
NA	8	5	3	

*Statistical significance is shown in bold*.

### Construction of Prognostic Gene Signature Related to Methyltransferase Complex in Diffuse Glioma

**Figure 2:**
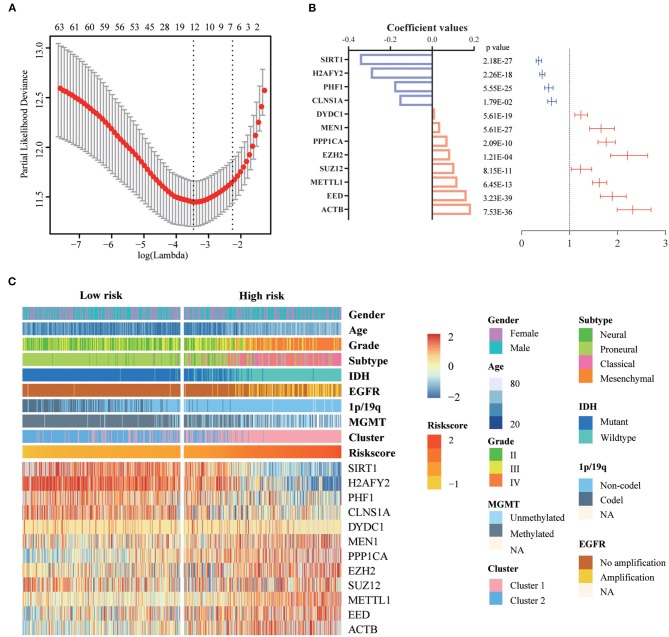
Identification of a 12-gene risk signature for OS by LASSO regression analysis in TCGA cohort. **(A)** Cross-validation for tuning parameter selection in the proportional hazards model. **(B)** Coefficient values and univariate Cox regression results for the 12 selected genes by LASSO. **(C)** Heatmap shows the association of risk scores and clinic pathological features based on the 12-gene risk signature. LASSO, Least Absolute Shrinkage and Selection Operator.

First, we identified 65 genes that were statistically related with OS of glioma patients in TCGA training cohort via univariate Cox regression analysis (*P* < 0.05). Then, a 12-gene signature was identified by LASSO regression algorithm (Figures 2A,B), and their enrichment components, biological function and protein-protein interaction have been annotated by STRING (Figure S2). The risk scores for patients were calculated with their expression level and regression coefficients (Figures 2B,C). Patients were separated into high-risk and low-risk group by using the median risk score as a threshold. As shown in Figure 2C and Table 2, patients in high-risk group were mainly older, high grade, classical or mesenchymal subtype, *IDH* wildtype, 1p/19q non-codel, *MGMT* promoter unmethylated and *EGFR* amplification (*P* < 0.0001), while patients in low-risk group represented younger, lower grade, proneural or neural subtype, *IDH* mutant, 1p/19q codel, *MGMT* promoter methylated and without *EGFR* amplification (*P* < 0.0001). The same regression coefficients were applied in CGGA validation cohort to calculate risk scores of patients. Consistently, the similar difference between the two groups have been shown in Table S2.

**Table 2:** Characteristics of patients in low and high risk scores in TCGA cohort.

**Characteristics**	***n***	**Risk score**		***P-*value**
		**Low**	**High**	
**Total Cases**	601	301	300	
**Age**				
**<**49	320	211	109	**<0.0001**
≥49	281	90	191	
**Gender**				
Male	351	178	173	0.7774
Female	250	123	127	
**Grade**				
II	211	168	43	**<0.0001**
III	236	131	105	
IV	154	2	152	
**Subtype**				
Neural	36	19	17	**<0.0001**
Proneural	381	279	102	
Classical	149	3	146	
Mesenchymal	35	0	35	
***IDH*** **status**				
Mutant	374	293	81	**<0.0001**
Wildtype	227	8	219	
**1p/19q status**				
Codel	149	142	7	**<0.0001**
Non-codel	223	150	73	
NA	229	9	220	
***MGMT*** **promoter status**				
Unmethylated	147	20	127	**<0.0001**
Methylated	420	281	139	
NA	34	0	34	
***EGFR*** **status**				
Amplification	106	1	105	**<0.0001**
No amplification	487	298	189	
NA	8	2	6	

*Statistical significance is shown in bold*.

### Prognostic Risk Scores of the 12-Gene Signature Is Related With Pathological Characteristics in Glioma

**Figure 3:**
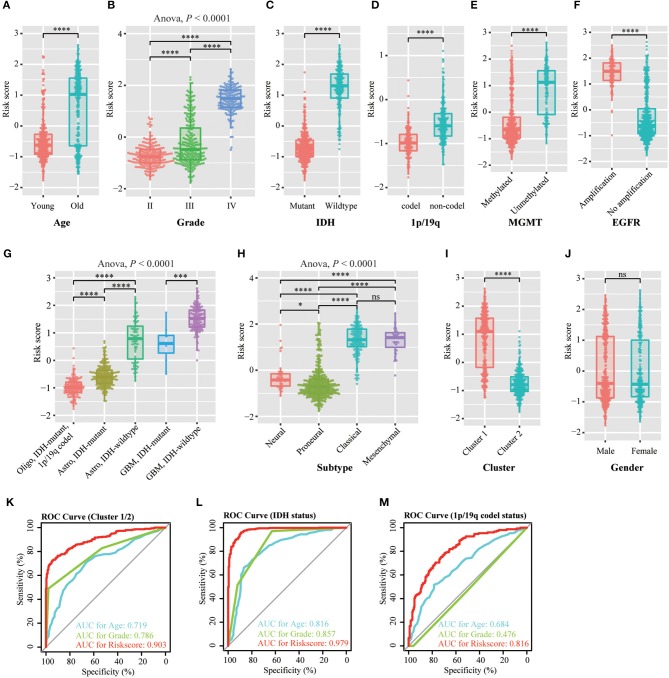
Association between the methyltransferase-related signature and other pathological features in TCGA cohort. **(A–J)** Distribution of the risk score in patients stratified by age **(A)**, WHO grade **(B)**, *IDH* status **(C)**, 1p/19q status **(D)**, *MGMT* promoter status **(E)**, *EGFR* status **(F)**, different pathological features **(G)**, TCGA subtype **(H)**, cluster **(I)** and gender **(J)**. **(K–M)** ROC curves showed the predictive efficiency of the risk signature, Cluster 1/2 subgroups **(K)**, *IDH* status **(L)** and 1p/19q status **(M)**. ****P < 0.0001; ***P < 0.001; *P < 0.05; ns, no significant.

We investigated the relationship between the risk scores of the 12-gene signature and the patients' pathological features. We observed that risk scores are significantly different between patients classified by age at diagnosis (*P* < 0.0001), WHO grade (*P* < 0.0001), *IDH* status (*P* < 0.0001), 1p/19q status (*P* < 0.0001), *MGMT* promoter status (*P* < 0.0001), *EGFR* status (*P* < 0.0001), different pathological features (*P* < 0.0001), TCGA subtype (*P* < 0.0001), and Cluster 1/2 (*P* < 0.0001), but not by gender in TCGA dataset (Figures 3A–J). The similar results were shown in CGGA dataset (Figures S3A–E,G–J), except *EGFR* status (Figure S3F). This is because the information of *EGFR* status in CGGA dataset is incomplete. Subsequently, we used the ROC curve to assess the specificity and sensitivity of risk scores in the prediction of pathological features by calculating the area under the curve (AUC) of risk score, age and grade. The risk score can perfectly predict Cluster 1/2 subgroups (AUC = 0.903 or 0.924), *IDH* mutant status (AUC = 0.979 or 0.925) and 1p/19q codel status (AUC = 0.816 or 0.723) in TCGA and CGGA datasets, which were higher than age and grade (Figures 3K–M, Figures S3K–M).

### Assessment of the Prognostic Value of the 12-Gene Signature

**Figure 4:**
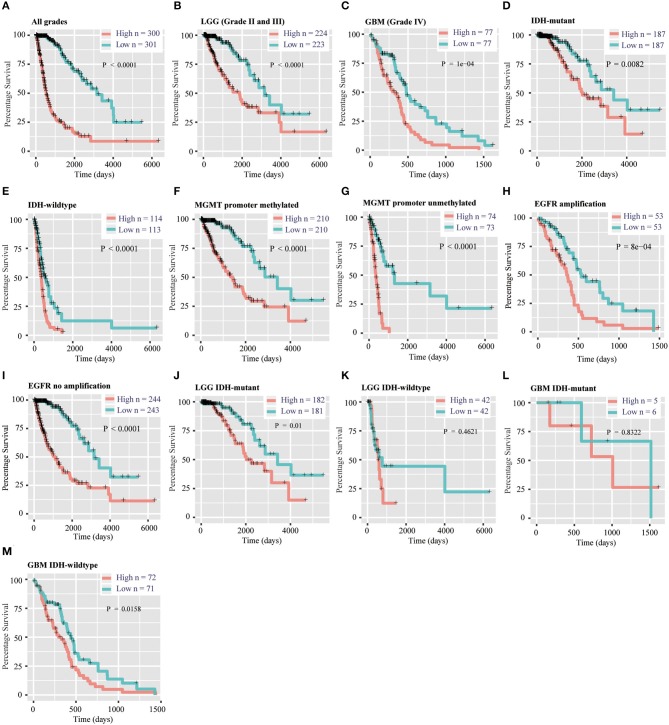
Prognostic significance of the 12-gene signature derived risk scores in TCGA cohort. **(A–C)** Prognosis efficiency of the 12-gene risk signature in all grades **(A)**, LGG **(B)** and GBM **(C)** in TCGA cohort. **(D–I)** Outcome prediction of the 12-gene signature in patients stratified by *IDH* status **(D,E)**, *MGMT* promoter status **(F,G)** and *EGFR* status **(H,I)** in TCGA cohort. **(J–K)** Kaplan–Meier survival curves for LGG patients with IDH-mutant **(J)** and IDH-wildtype **(K)**, classified into two groups based on 12-gene signature derived risk scores. **(L–M)** Kaplan–Meier survival curves for GBM patients with IDH-mutant **(L)** and IDH-wildtype **(M)**, classified into two groups based on 12-gene signature derived risk scores. LGG, lower-grade glioma; GBM, glioblastoma; OS, overall survival.

To evaluate the prognostic value of the signature, patients were separated into high-risk and low-risk group by using the median risk score as a threshold. By Kaplan–Meier survival analysis, the overall survival time of patients in the low-risk group were statistically longer than that in the high-risk group in the TCGA RNA-seq cohort (*P* < 0.0001, Figure 4A). Then, we explored the prognostic value of the 12-gene signature in patients with lower-grade glioma (LGG, WHO grade II and III) and GBM (WHO grade IV), respectively. High-risk scores in patients with LGG and GBM were significantly associated with lower overall survival time (*P* < 0.0001, *P* = 1e-4, Figures 4B,C). Then, we separately stratified patients by *IDH* status, *MGMT* promoter status and *EGFR* status, and investigated the prognostic value of this signature in subgroups. The comparable results were demonstrated in stratified patients (all *P* < 0.01, Figures 4D–I). Consistently, the prognostic significance of this 12-gene signature was verified in CGGA validation cohort (all *P* < 0.05, Figures S4A–G, I), except *EGFR* amplification group which only contain four samples (*P* = 0.3173, Figure S4H). In further analysis, the results showed that high risk score had a worse prognosis in LGG with IDH-mutant subgroup in both cohorts (*P* = 0.01 and 0.0026, Figure 4J, Figure S4J), but no significant difference in GBM IDH-mutant subgroup (*P* > 0.05, Figure 4L, Figure S4L). In LGG IDH-wildtype (Figure 4K, Figure S4K) and GBM IDH-wildtype (Figure 4M, Figure S4M) subgroups, we did not get consensus results in TCGA and CGGA RNA-seq datasets, which probably due to the small sample size or racial differences.

### The Risk Score of 12-Gene Signature Is an Independent Prognostic Indicator

**Figure 5:**
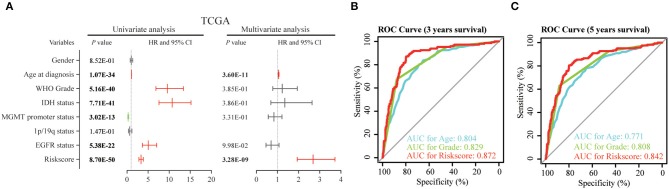
Univariate and multivariate analysis shows prognostic value of 12-gene signature in the TCGA dataset. **(A)** Univariate and multivariate Cox regression analyses of the association between clinic pathological factors and OS of patients in the TCGA dataset. **(B,C)** The receiver operator characteristic (ROC) curves to predict the sensitivity and specificity of 3 and 5 years survival according to the 12-gene signature derived risk scores in TCGA cohort. OS, overall survival.

Then, we used uni- and multi-variate Cox analysis to evaluate whether the risk score is an independent prognostic indicator. As shown in Table 3 and Figure 5A, factors including age at diagnosis, WHO grade, IDH status, MGMT promoter status, EGFR status and risk score were statistically related with the overall survival (OS) of glioma patients. Among them, age at diagnosis (HR, 1.053; 95% CI, 1.037–1.069; *P* = 3.60E-11) and risk score (HR, 2.684; 95% CI, 1.935–3.722; *P* = 3.28E-09) were independent prognostic indicators for OS in patients with gliomas in TCGA dataset (Table 3, Figure 5A). Similar results were found in the CGGA validation dataset; WHO grade (HR, 2.335; 95% CI, 1.412–3.861; *P* = 9.51E-04) and the risk score (HR, 1.936; 95% CI, 1.365–2.745; *P* = 2.12E-04) were independent prognostic indicators for OS in patients with gliomas (Table S3, Figure S5A). Compared to the traditional factors age (AUC = 0.804 or 0.771) and grade (AUC = 0.829 or 0.808), the risk score (AUC = 0.872 or 0.842) showed striking prognostic predictive efficiency for 3 and 5 years survival rates in TCGA dataset (Figures 5B,C). Consistently, The AUC of risk score (AUC = 0.789 or 0.778) was substantially higher than that of age (AUC = 0.654 or 0.635) and grade (AUC = 0.778 or 0.751) in CGGA validation cohort (Figures S5B,C). Taken together, the above results indicated that the risk score of 12-gene signature was an independent prognostic indicator for OS in diffuse glioma patients.

**Table 3:** Univariate and multivariate Cox regression analysis of clinical pathologic features for OS in TCGA cohort.

**Variables**	**Univariate Cox Regression**			**Multivariate Cox Regression**		
	**HR**	**95% CI**	***P*-value**	**HR**	**95% CI**	***P*-value**
Gender	1.028	0.765–1.382	8.52E−01			
Age at diagnosis	1.076	1.064–1.089	1.07E−34	1.053	1.037–1.069	3.60E−11
WHO Grade	9.581	6.857–13.388	5.16E−40	1.226	0.775–1.938	3.85E−01
*IDH* status	10.711	7.568–15.159	7.71E−41	1.346	0.687–2.638	3.86E−01
*MGMT* promoter status	0.297	0.214–0.411	3.02E−13	0.828	0.565–1.212	3.31E−01
1p/19q status	0.639	0.348–1.170	1.47E−01			
*EGFR* status	5.056	3.637–7.029	5.38E−22	0.7	0.458–1.070	9.98E−02
Risk score	3.317	2.831–3.886	8.70E−50	2.684	1.935–3.722	3.28E−09

*P **<** 0.05 was considered statistically significant. Gender (female vs. male); WHO grade (lower grade vs glioblastoma); IDH status (mutant and wildtype); 1p/19q status (codeletion and non-codeletion); MGMT promoter status (methylated and unmethylated); EGFR status (amplification and no amplification). CI, confidence interval; HR, hazard ratio; IDH, isocitrate dehydrogenase; MGMT, O6-methylguanine-DNA methyltransferase*.

### Biological Characteristics and Pathway Analysis of 12-Gene Signature

**Figure 6:**
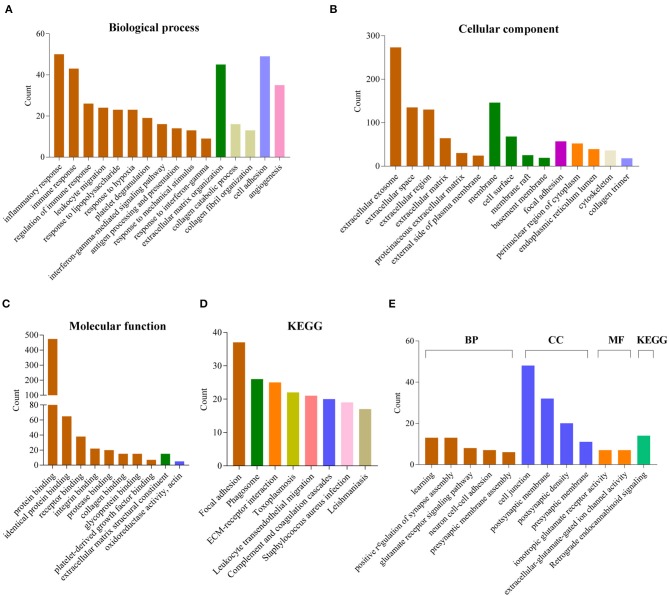
Functional annotation related to the 12-gene signature. **(A–D)** Functional annotation of genes positively correlated with the risk score using GO terms of biological process **(A)**, cellular component **(B)**, molecular function **(C)** and KEGG pathway **(D)** by DAVID. **(E)** Functional annotation of genes negatively correlated with the risk score using GO terms and KEGG pathway by DAVID.

To explore the potential function of 12 gene signature, we first used Pearson correlation analysis to determine genes that were statistically positively (*R* > 0.5, *P* < 0.0001) or negatively (*R* < – 0.5, *P* < 0.0001) related with the risk sore of gene signature in TCGA and CGGA datasets. Totally, 867 positively and 787 negatively correlated genes were identified in these two data sets. Then their biological characteristics and pathway were annotated by GO terms and KEGG pathway (*P* < 0.05, Benjamini and Hochberg method). Positively related genes were mainly involved in biological process (BP), including immune response (such as inflammatory response, interferon-gamma-mediated signaling pathway, response to interferon-gamma), extracellular matrix organization, cell adhesion, angiogenesis (Figure 6A). The most enriched cellular component (CC) terms were extracellular component (extracellular exosome, extracellular space, extracellular matrix), membrane system (membrane, cell surface, membrane raft) and focal adhesion (Figure 6B). The most enriched molecular function (MF) terms were protein binding (Figure 6C). The most enriched pathways were focal adhesion, phagosome, ECM-receptor interaction, leukocyte transendothelial migration, complement and coagulation cascades by KEGG pathway analysis (Figure 6D).

In contrast, negatively related genes were mainly involved in BP terms, including learning, positive regulation of synapse assembly, glutamate receptor signaling pathway, neuron cell-cell adhesion, presynaptic membrane assembly. The enriched CC terms were cell junction, postsynaptic membrane, postsynaptic density, presynaptic membrane. The enriched MF terms were ionotropic glutamate receptor activity, extracellular-glutamate-gated ion channel activity. The enriched KEGG pathway was retrograde endocannabinoid signaling (Figure 6E).

Moreover, the GSEA analyses showed consistent results. The high-risk groups were enriched in immune response (such as adaptive immune response based on somatic recombination of immune receptors built from immunoglobulin superfamily domains, lymphocyte mediated immunity, complement and coagulation cascades), extracellular structure organization, ECM-receptor interaction, focal adhesion (Figure S6).

### Discussion

In this study, for the first time, we found methyltransferase-related genes could distinguish glioma patients into two clusters which showed significant differences in clinical and molecular features. Then, we built a methyltransferase-related gene signature that could classify patients into high and low-risk group by an elastic net regression Cox model (32). Most of the genes in the signature are mainly enriched in methyltransferase complex and nucleoplasm (Figure S2A). And they can bind with DNA or/and protein (Figure S2B). Protein-protein interaction analysis showed that five genes (EZH2, SUZ12, EED, PHF1 and SIRT1) are core components of PRC2 complex, which execute transcriptional inhibition via catalyzing H3K27me3 (Figure S2C). EZH2 protein expression is significantly higher in GBM, and it is a negative prognostic factor in GBM (13, 33, 34). EZH2 has been reported as an oncogene and is involved in several glioma cell processes, including cell cycle, invasion, GSC maintenance, drug and radiotherapy resistance, etc (35). The expression of SUZ12 protein was significantly upregulated in tumor compared with its adjacent brain tissue by western blot and immunohistochemistry analysis (36). miR-128, miR-105 and miR-767-5p are suppressors for glioma cell malignancy by targeting *SUZ12* (36–38). The function of SIRT1 in glioma is complicated. On the one hand, *SIRT1* knockdown significantly inhibited glioma cell proliferation, migration, invasion, promoted its apoptosis and potentiated TMZ toxicity (39–41). On the other hand, the expression of SIRT1 in GBM is significantly lower than normal brain tissue (42, 43). Up-regulation of SIRT1 by genetic modification or treatment of melatonin significantly attenuated the adhesion molecular VCAM-1 and ICAM-1 expression in GBM, which modulated the monocytes interaction with GBM (43). SIRT1 activator SRT2183 suppresses glioma cell growth and destroyed neurospheres *in vitro* (44). The effect of SIRT1 on glioma progression still needs more *in vivo* experiments to verify. MEN1 expression was activated in 44.4% of adult gliomas and predicted poor prognosis of patients with glioma (45). Importantly, MEN1 inhibitors significantly decreased the proliferation of adult glioma cells (45). CLNS1A is a co-factor of methyltransferase PRMT5. They are components of methylosome, a multi-subunit complex which modifies specific Sm-proteins to facilitate small ribonucleoprotein (snRNP) assembly (46, 47). *CLNS1A* knockdown increased sensitivity to PRMT5 inhibitor EPZ015666 in malignant glioma, which may due to the reducing of splicing capacity (48). The protein expression of PPP1CA is high in GBM, but it showed no correlation with prognosis in all GBMs or on stratification based on IDH1 or ATRX expression (49). However, PPP1CA expression is associated with poor prognosis in p53 expressing GBMs (49). METTL1 mediated tRNA and microRNA processing via N^7^-methylguanosine (m^7^G) methylation (50, 51). In addition to the essential role of METTL1 in embryonic stem cell self-renewal and differentiation (52), it is elevated in hepatocellular carcinoma (HCC) and shows carcinogenic activity through PTEN/AKT signaling pathway (53). DYDC1 is a component of MLL3/4 complex, which can methylate lysine-4 of histone H3. Collectively, the expression of EZH2 (33), SUZ12 (36), SIRT1 (42, 43), MEN1 (45) and PPP1CA (49) at RNA levels are consistent with protein levels in previous reports, indicating that analysis based on RNA sequencing data can be verified by other techniques. Fifty percentage (6/12) of genes in this signature have been reported to participate the progression of glioma, which verified the value of methyltransferase-related signature.

Based on TCGA training set and CGGA validation set, we observed that the risk scores are much higher in WHO grade IV, *IDH* wildtype, 1p/19q non-codel, *MGMT* promoter unmethylated, *EGFR* amplification and worse TCGA subtypes (classical and mesenchymal). It implies that this methyltransferase-related gene signature may predict the prognosis of patients with glioma. Next, we evaluated the 12-gene risk signature prognostic value in patients with glioma. This methyltransferase-related signature was a mighty prognostic indicator regardless of WHO grade, *IDH* status, *MGMT* promoter status and *EGFR* status in both datasets. After stratified patients into four subgroups by WHO grade and *IDH* status, it only predicted the prognosis of LGG IDH-mutant diffuse glioma patients, which may due to the small sample size of other groups. Moreover, we found the risk score of methyltransferase-related signature was an independent prognostic indicator for OS in diffuse glioma patients when considering several clinical and molecular characteristics. And it is could better predict the prognosis of glioma than the traditional factors “age” and “grade.” These analysis indicated that this signature is a mighty prognostic indicator, and it might be used to classify patients and guide targeted therapy in the future.

For biological characteristics and pathway analysis of 12-gene signature, the significantly correlated genes (|R| > 0.5, *P* < 0.0001) were performed GO and KEGG analysis. Results showed that positively correlated genes are significantly enriched in BP, such as immune response, extracellular matrix organization, cell adhesion and angiogenesis. For KEGG, these genes were enriched in focal adhesion, phagosome, ECM-receptor interaction, leukocyte transendothelial migration, complement and coagulation cascades. These results indicated that the high risk score group may affect the glioma progression by affecting these biological processes or pathways. Meanwhile, the negatively correlated genes were closely related to GO terms of the normal nervous system, such as learning, positive regulation of synapse assembly, glutamate receptor signaling pathway, neuron cell-cell adhesion, presynaptic membrane assembly, ionotropic glutamate receptor activity, extracellular-glutamate-gated ion channel activity. It indicated that the low risk score group were more similar to the normal nervous system. GSEA analyses is consistent with the above results.

### Conclusions

We identified that the methyltransferase genes could classify the glioma patients with different clinical and molecular characteristics. We then built a 12-gene risk signature, which was strongly associated with pathological features in glioma. Moreover, the risk score of this signature was an independent prognostic indicator. Furthermore, the biological process and pathway related with this risk signature had been annotated. Our study provides new understanding of methyltransferase in the carcinogenesis and development of glioma. It provided important evidence for future application of methyltransferase inhibitor in glioma therapies. However, our study is based on RNA sequencing technology for large-scale detection of gene expression at the RNA level. Therefore, the ability of this signature to predict prognosis should be retested in further research by other techniques or validated in pathological sections, primary gliomas cells before clinical application.

## Data Availability Statement

Publicly available datasets were analyzed in this study. These data can be found here: http://www.cgga.org.cn/; https://cancergenome.nih.gov/.

## Author Contributions

YZ and YL provided equal contributions to research design, data analysis and article writing. HL, ZZ and FW participated in data downloading and preliminary analysis. FZ revised the manuscript. The final manuscript has been read and approved by all authors.

### Conflict of Interest

The authors declare that the research was conducted in the absence of any commercial or financial relationships that could be construed as a potential conflict of interest.
