# Supplementary material for: Clinical and Biological Significances of a Methyltransferase-Related Signature in Diffuse Glioma
Source: Front Oncol. 2020 Apr 20;10:508. doi: 10.3389/fonc.2020.00508 (PMC7185060; doi:10.3389/fonc.2020.00508)
Supplement: Supplementary file 1 [file Data_Sheet_1.docx]

**
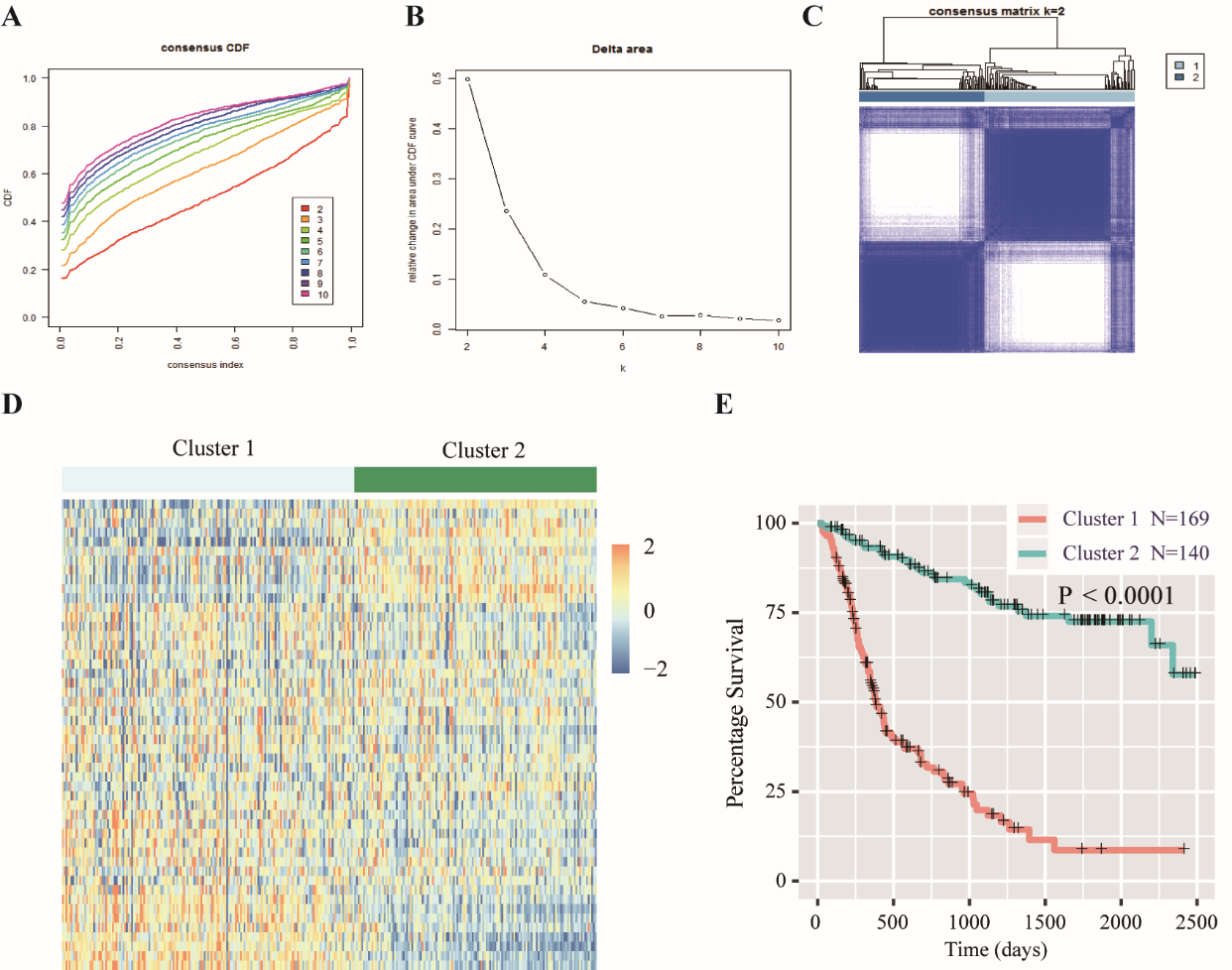
**

**Figure S1.** Methyltransferase-related genes could classify the clinical and molecular features of gliomas. (A) Consensus clustering cumulative distribution function (CDF) for k = 2 to k = 10. (B) Relative change in area under CDF curve according to various k values. (C) Consensus clustering matrix of 309 samples from CGGA dataset for k = 2. (D) Heat map of two clusters defined by the top 50 variable expression genes. (E) Survival analysis of patients in Cluster 1 and Cluster 2 in CGGA cohort.

**
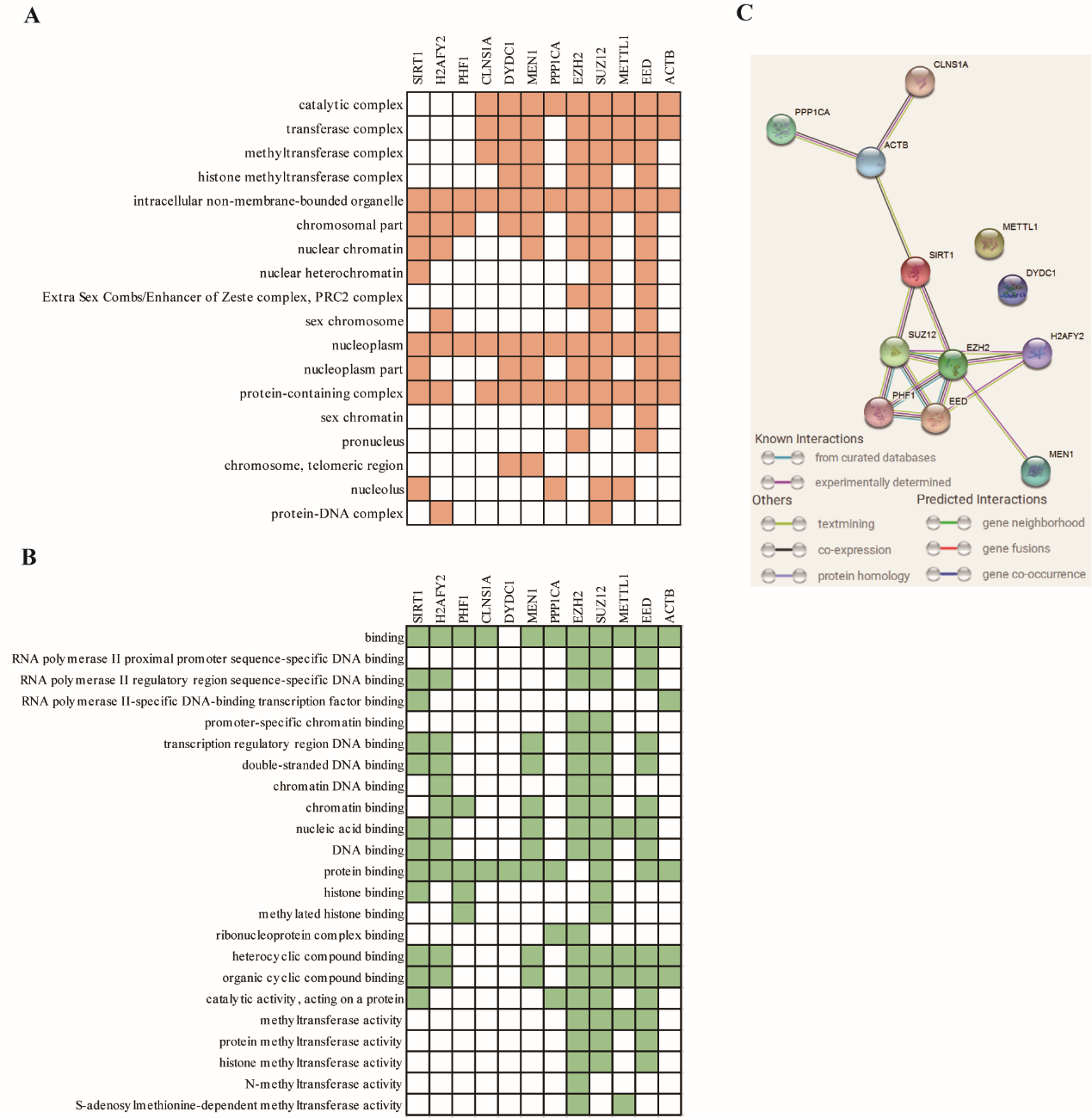
Figure S2.** Functional enrichments of the 12 methyltransferase-related genes in prognostic signature has been analyzed by STRING. (A) Cellular component. (B) Biological function. (C) Protein-protein interaction network.

**
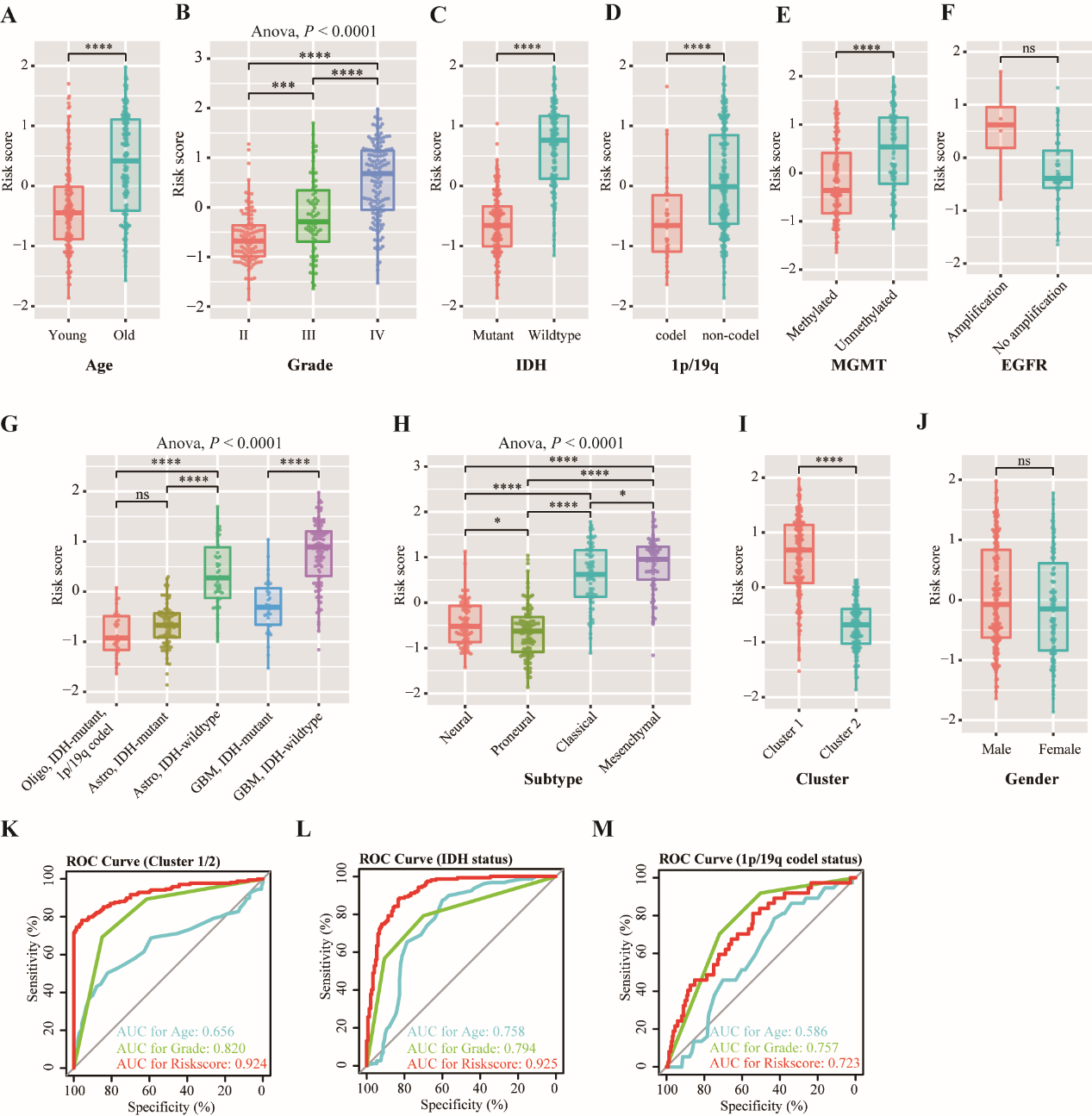
 Figure S3.** Association between the methyltransferase-related signature and other pathological features in CGGA cohort. (A-J) Distribution of the risk score in patients stratified by age (A), WHO grade (B), *IDH* status (C), 1p/19q status (D), *MGMT* promoter status (E), *EGFR* status (F), different pathological features (G), TCGA subtype (H), cluster (I) and gender (J). (K-M) ROC curves showed the predictive efficiency of the risk signature, Cluster 1/2 subgroups (K), *IDH* status (L) and 1p/19q status (M). *****P* < 0.0001; ****P* < 0.001; ***P* < 0.01; **P* < 0.05; ns, no significant.

**
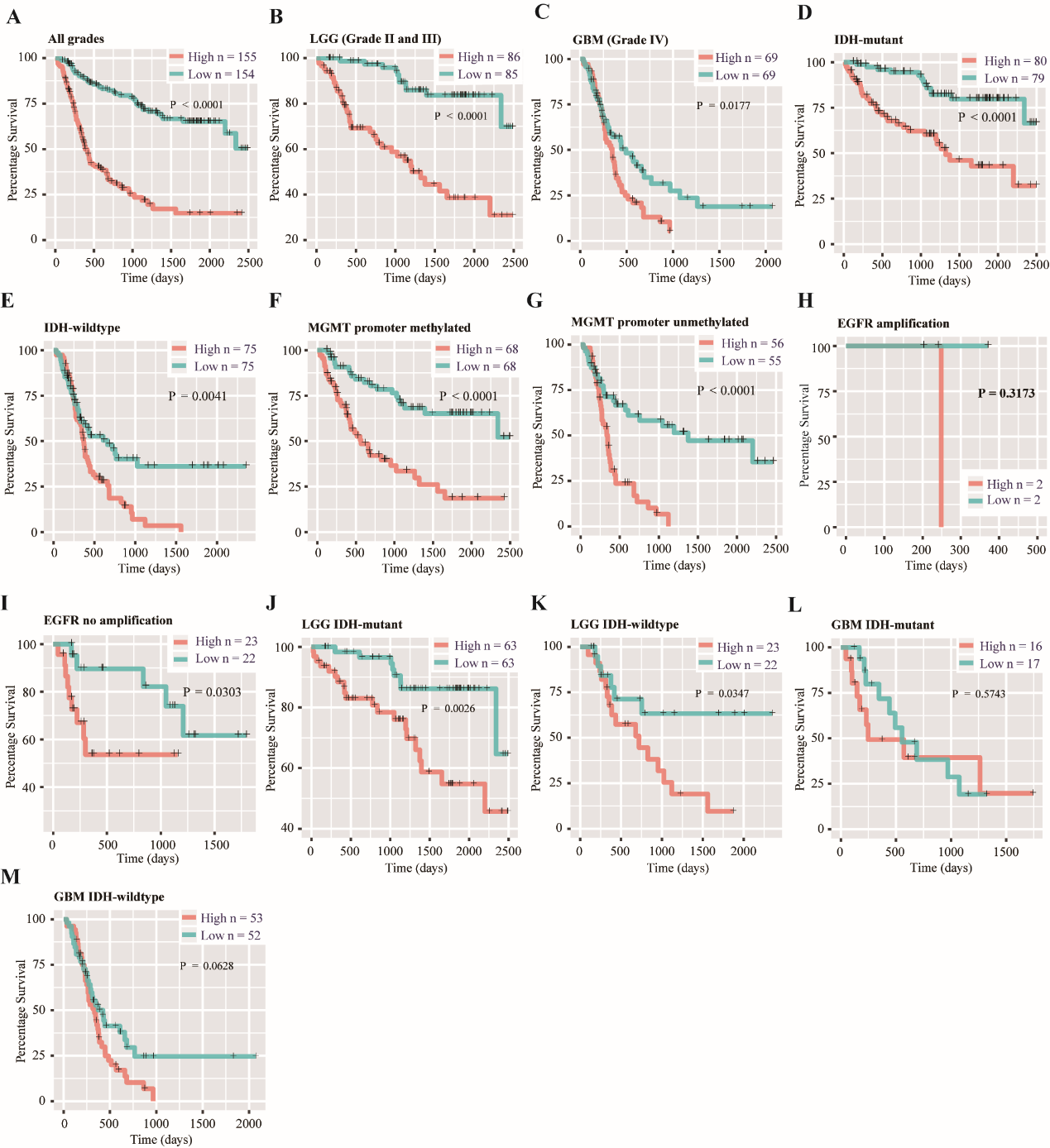
Figure S4.** Prognostic significance of the 12-gene signature derived risk scores in CGGA cohort. (A-C) Prognosis efficiency of the 12-gene risk signature in all grades (A), LGG (B) and GBM (C) in CGGA cohorts. (D-I) Outcome prediction of the 12-gene signature in patients stratified by *IDH* status (D and E), *MGMT* promoter status (F and G) and *EGFR* status (H and I) in CGGA cohort. (J-K) Kaplan-Meier survival curves for LGG patients with IDH-mutant (J) and IDH-wild type (K), classified into two groups based on 12-gene signature derived risk scores. (L-M) Kaplan-Meier survival curves for GBM patients with IDH-mutant (L) and IDH-wildtype (M), classified into two groups based on 12-gene signature derived risk scores. LGG, lower-grade glioma; GBM, glioblastoma; OS, overall survival.


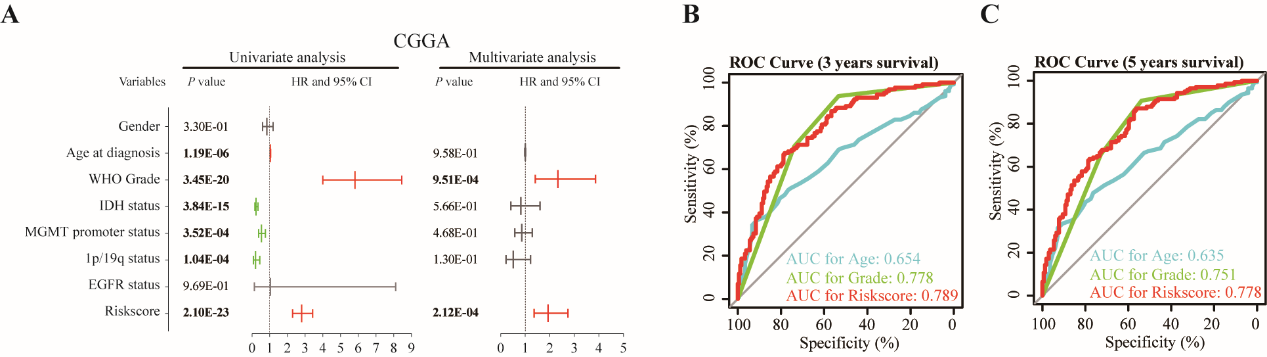


**Figure S5.** Univariate and multivariate analysis shows prognostic value of 12-gene signature in the CGGA datasets. (A) Univariate and multivariate Cox regression analyses of the association between clinic pathological factors and OS of patients in the CGGA datasets. (B-C) The receiver operator characteristic (ROC) curves to predict the sensitivity and specificity of 3 and 5 years survival according to the 12-gene signature derived risk scores in CGGA cohort. OS, overall survival.

**
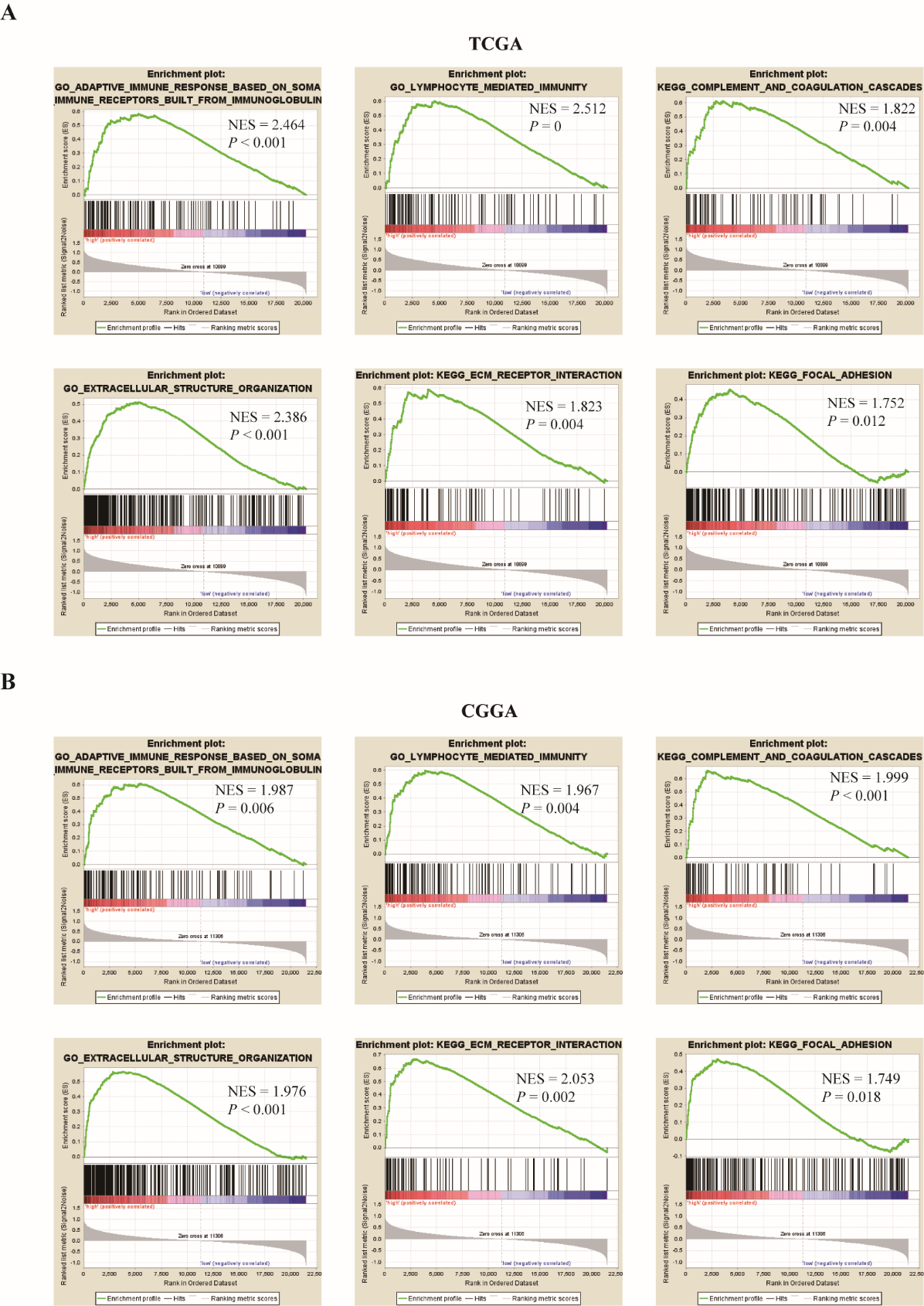
Figure S6.** Gene set enrichment analysis (GSEA) analysis based on the median value of risk score in the TCGA (A) and CGGA (B) dataset. NES, normalized enrichment score.

**Table S1.** Characteristics of patients in cluster 1 and cluster 2 in CGGA cohort.

| **Characteristics** | | **n** | **Cluster 1** | **Cluster 2** | ***P* value** | |
| --- | --- | --- | --- | --- | --- | --- |
| **Total Cases** | | 309 | 169 | 140 |  | |
| **Age** | |  |  |  |  | |
|  | **<** 43 | 153 | 65 | 88 | **< 0.0001** | |
|  | ≥ 43 | 156 | 104 | 52 |  | |
| **Gender** | |  |  |  |  | |
|  | Male | 194 | 114 | 80 | 0.0804 | |
|  | Female | 115 | 55 | 60 |  | |
| **Grade** | |  |  |  |  | |
|  | II | 104 | 18 | 86 | **< 0.0001** | |
|  | III | 67 | 34 | 33 |  | |
|  | IV | 138 | 117 | 21 |  | |
| **Subtype** | |  |  |  |  | |
|  | Neural | 76 | 14 | 62 | **< 0.0001** | |
|  | Proneural | 99 | 37 | 62 |  | |
|  | Classical | 69 | 60 | 11 |  | |
|  | Mesenchymal | 65 | 58 | 65 |  | |
| ***IDH* status** | |  |  |  |  | |
|  | Mutant | 159 | 46 | 113 | **< 0.0001** | |
|  | Wildtype | 150 | 123 | 27 |  | |
| **1p/19q status** | |  |  |  |  | |
|  | Codel | 37 | 9 | 28 | **0.0003** | |
|  | Noncodel | 221 | 127 | 94 |  | |
|  | NA | 51 | 33 | 18 |  | |
| ***MGMT* promoter status** | | | | | |  |
|  | Unmethylated | 111 | 76 | 35 | **0.0002** | |
|  | Methylated | 136 | 70 | 66 |  | |
|  | NA | 62 | 23 | 39 |  | |
| ***EGFR* status** | | | | | | |
|  | Amplification | 4 | 4 | 0 | **0.1855** | |
|  | No amplification | 45 | 24 | 21 |  | |
|  | NA | 260 | 141 | 119 |  | |

**Table S2.** Characteristics of patients in low and high risk score in CGGA cohort.

| **Characteristics** | | **n** | **Risk score** | | ***P* value** |
| --- | --- | --- | --- | --- | --- |
|  |  |  | **Low** | **High** |  |
| **Total Cases** | | 309 | 154 | 155 |  |
| **Age** | |  |  |  |  |
|  | **<** 43 | 153 | 107 | 46 | **< 0.0001** |
|  | ≥ 43 | 156 | 47 | 109 |  |
| **Gender** | |  |  |  |  |
|  | Male | 194 | 93 | 101 | 0.4533 |
|  | Female | 115 | 61 | 54 |  |
| **Grade** | |  |  |  |  |
|  | II | 104 | 85 | 19 | **< 0.0001** |
|  | III | 67 | 38 | 29 |  |
|  | IV | 138 | 31 | 107 |  |
| **Subtype** | |  |  |  |  |
|  | Neural | 76 | 53 | 23 | **< 0.0001** |
|  | Proneural | 99 | 83 | 16 |  |
|  | Classical | 69 | 13 | 64 |  |
|  | Mesenchymal | 65 | 5 | 65 |  |
| ***IDH* status** | |  |  |  |  |
|  | Mutant | 159 | 133 | 26 | **< 0.0001** |
|  | Wildtype | 150 | 21 | 129 |  |
| **1p/19q status** | |  |  |  |  |
|  | Codel | 37 | 29 | 8 | **0.0010** |
|  | Noncodel | 221 | 101 | 120 |  |
|  | NA | 51 | 24 | 27 |  |
| ***MGMT* promoter status** | | | | | |
|  | Unmethylated | 111 | 31 | 80 | **< 0.0001** |
|  | Methylated | 136 | 79 | 57 |  |
|  | NA | 62 | 44 | 18 |  |
| ***EGFR* status** | | | | | |
|  | Amplification | 4 | 1 | 3 | **0.0342** |
|  | No amplification | 45 | 30 | 15 |  |
|  | NA | 260 | 123 | 137 |  |

**Table S3.** Univariate and multivariate Cox regression analysis of clinical pathologic features for OS in CGGA cohort.

| Variables | Univariate Cox Regression | | | Multivariate Cox Regression | | |
| --- | --- | --- | --- | --- | --- | --- |
|  | HR | 95% CI | *P* value | HR | 95% CI | *P* value |
| Gender | 0.843 | 0.597-1.189 | 3.30E-01 |  |  |  |
| Age at diagnosis | 1.038 | 1.022-1.053 | 1.19E-06 | 1.000 | 0.983-1.019 | 9.58E-01 |
| WHO Grade | 5.812 | 3.996-8.455 | 3.45E-20 | 2.335 | 1.412-3.861 | 9.51E-04 |
| *IDH* status | 0.229 | 0.159-0.331 | 3.84E-15 | 0.823 | 0.422-1.602 | 5.66E-01 |
| *MGMT* promoter status | 0.529 | 0.374-0.750 | 3.52E-04 | 0.860 | 0.573-1.291 | 4.68E-01 |
| 1p/19q status | 0.196 | 0.086-0.447 | 1.04E-04 | 0.519 | 0.222-1.213 | 1.30E-01 |
| *EGFR* status | 1.041 | 0.134-8.106 | 9.69E-01 |  |  |  |
| Risk score | 2.799 | 2.286-3.427 | 2.10E-23 | 1.936 | 1.365-2.745 | 2.12E-04 |
| *P* **<** 0.05 was considered statistically signiﬁcant. Gender (female vs male); WHO grade (lower grade vs glioblastoma); *IDH* status (mutant and wildtype);1p/19q status (codeletion and non-codeletion); *MGMT* promoter status (methylated and unmethylated); *EGFR* status (amplification and no amplification). Abbreviations: CI, conﬁdence interval; HR, hazard ratio; IDH, isocitrate dehydrogenase; MGMT, O6-methylguanine-DNA methyltransferase. | | | | | | |
